# Chronic Nicotine Exposure Initiated in Adolescence and Unpaired to Behavioral Context Fails to Enhance Sweetened Ethanol Seeking

**DOI:** 10.3389/fnbeh.2017.00153

**Published:** 2017-08-17

**Authors:** Aric C. Madayag, Kyle S. Czarnecki, Lynde M. Wangler, Donita L. Robinson

**Affiliations:** ^1^Bowles Center for Alcohol Studies, University of North Carolina Chapel Hill, NC, United States; ^2^Department of Pharmacology, University of North Carolina Chapel Hill, NC, United States; ^3^Department of Psychiatry, University of North Carolina Chapel Hill, NC, United States

**Keywords:** alcohol use disorder, adolescent, addiction, cigarette smoking, tobacco

## Abstract

Nicotine use in adolescence is pervasive in the United States and, according to the Gateway Hypothesis, may lead to progression towards other addictive substances. Given the prevalence of nicotine and ethanol comorbidity, it is difficult to ascertain if nicotine is a gateway drug for ethanol. Our study investigated the relationship between adolescent exposure to nicotine and whether this exposure alters subsequent alcohol seeking behavior. We hypothesized that rats exposed to nicotine beginning in adolescence would exhibit greater alcohol seeking behavior than non-exposed siblings. To test our hypothesis, beginning at P28, female rats were initially exposed to once daily nicotine (0.4 mg/kg, SC) or saline for 5 days. Following these five initial injections, animals were trained to nose-poke for sucrose reinforcement (10%, w/v), gradually increasing to sweetened ethanol (10% sucrose; 10% ethanol, w/v) on an FR5 reinforcement schedule. Nicotine injections were administered after the behavioral sessions to minimize acute effects of nicotine on operant self-administration. We measured the effects of nicotine exposure on the following aspects of ethanol seeking: self-administration, naltrexone (NTX)-induced decreases, habit-directed behavior, motivation, extinction and reinstatement. Nicotine exposure did not alter self-administration or the effectiveness of NTX to reduce alcohol seeking. Nicotine exposure blocked habit-directed ethanol seeking. Finally, nicotine did not alter extinction learning or cue-induced reinstatement to sweetened ethanol seeking. Our findings suggest that nicotine exposure outside the behavioral context does not escalate ethanol seeking. Further, the Gateway Hypothesis likely applies to scenarios in which nicotine is either self-administered or physiologically active during the behavioral session.

## Introduction

Conventional cigarette use among youth is in steady decline; however, novel modes of consumption have arisen and are more readily available, potentially leading to increased levels of nicotine exposure. In 2016, according to the National Institutes of Health “Monitoring the Future Survey 2016”, the percent of youth using conventional cigarettes in the past month among 8th, 10th and 12th grades was 2.6%, 4.9% and 10.5% respectively. Conversely, e-cigarette use encompassed 6.2%, 11.0% and 12.5% at the same grade level. These statistics argue that consumption of nicotine by conventional tobacco sources, e-cigarettes, or smokeless tobacco may pose a health threat to the future of the youth in the United States. In turn, alcohol use among adolescents is widespread, with over 35% of youth grades 8–12 in the United States reporting regular consumption and approximately 15% reporting heavy drinking or binge-drinking episodes (Bachman, 2002). Binge-level alcohol consumption is associated with elevated risk for numerous adverse outcomes including drug abuse susceptibility (Brown et al., 2000). Despite the prevalence of nicotine and ethanol use during adolescence, and their comorbidity, it is unknown if the two substances interact directly or through long-term physiological mechanisms to elicit a synergistic behavioral response. With apparent ease of access to nicotine and ethanol, the aim of this study was to determine if adolescents that are exposed to nicotine are also more susceptible to escalate ethanol seeking.

Preclinical studies suggest that adolescent animals are prone to nicotine and alcohol use. Compared to adults, adolescent rodents exhibit a higher preference for nicotine (Torres et al., 2009; Nesil et al., 2011) and higher rewarding effects of nicotine (Shram and Lê, 2010), though lower operant responding for nicotine (Shram et al., 2008; Schassburger et al., 2016). In turn, adolescent rodents show greater propensity to self-administer ethanol (Tambour et al., 2008; Walker and Ehlers, 2009; Doherty and Gonzales, 2015; Serlin and Torregrossa, 2015) and a higher preference for ethanol over water control (Truxell et al., 2007; Melendez, 2011). These findings collectively suggest adolescents are more prone to substance use and potential abuse, which could render them susceptible to addiction in adulthood. The Common Liability Hypothesis (Palmer et al., 2012) posits that underlying genetic or environmental predispositions are the important factors leading to addiction, and the developmental preference for addictive drugs during adolescence may fall into a “predisposition,” albeit developmentally limited. Alternatively, the Gateway Hypothesis (Kandel et al., 1992; Degenhardt et al., 2010) theorizes that drug exposure “primes” the brain in such a way as to promote later addiction to a variety of drugs. Discerning the respective contributions of drug exposure, genetic, and environmental factors has proved to be difficult in human studies due to the inability to manipulate such variables ethically.

Nicotine has been suggested as a potent gateway drug (Kandel and Kandel, 2014). Specifically, animal studies found that nicotine produces molecular, physiological and behavioral changes to render higher susceptibility to later cocaine use (Levine et al., 2011). However, it is not clear whether nicotine use during adolescence—when many people initiate drug use—is associated with more profound “gateway” effects towards ethanol. Studies indicate that animals will escalate ethanol self-administration when it is co-administered with nicotine (Lárraga et al., 2017), suggesting that nicotine exposure escalates ethanol seeking. In Pavlovian-based tasks, when given concurrently with the initiation of a conditioned approach session where ethanol is the unconditioned stimulus, nicotine enhances approach behavior, though not when administered repeatedly in days leading up to the beginning of behavior sessions (Maddux and Chaudhri, 2017). Moreover, we and others have observed that nicotine is sufficient to elevate conditioned responding to cues predicting sucrose or water reward (Olausson et al., 2004a,b; Stringfield et al., 2017), suggesting non-specific elevation of conditioned responding. It is unknown if exposure to nicotine *prior to* ethanol self-administration sessions can lead to such an escalation of ethanol seeking, as would be indicated by the Gateway Hypothesis for nicotine (Kandel and Kandel, 2014).

Consistent with the Gateway Hypothesis for nicotine, we hypothesized that exposure to nicotine in adolescence would increase the rewarding and motivational properties of ethanol. To test this, we exposed adolescent female rats to daily nicotine injections and tested operant responding for sweetened ethanol under multiple conditions to ascertain ethanol seeking, habit-directed behavior, motivation for ethanol and extinction-reinstatement. Nicotine exposure continued through the operant training and testing. Notably, operant sessions and nicotine administration were separated in time so that any changes in behavior between groups would be due to nicotine exposure and associated neuroplasticity rather than acute effects.

## Materials and Methods

### Subjects

Eighteen Sprague-Dawley female rats were bred at the University of North Carolina at Chapel Hill and maintained under a 12:12 h light:dark cycle, lights on from 07:00 to 19:00. Animals were weaned into pair-housed cages on postnatal day (P) 21 with *ad libitum* access to water and standard chow except when indicated. All experimental procedures were performed during the light cycle between 09:00 and 12:00. This study was carried out in accordance with the recommendations of University of North Carolina Division of Laboratory Animal Medicine. The protocol was approved by the Institutional Animal Care and Use Committee of the University of North Carolina. The dataset consisted of four litters grouped into three behavior cohorts. The following is a breakdown of experimental group distribution: cohort 1, 4 saline and 4 nicotine; cohort 2, 2 saline and 4 nicotine; cohort 3, 2 saline and 2 nicotine. Every animal performed the entirety of behavior experiments.

### Drugs

Nicotine hydrogen tartrate (Sigma Aldrich, St. Louis, MO, USA) was dissolved in 0.9% saline and pH adjusted to 7.0 ± 0.2 via NaOH solution. Nicotine was administered via subcutaneous injection at 0.4 mg/kg, calculated using the free base form (Palmatier et al., 2013; Stringfield et al., 2017). Ethanol (95% Decon Labs, King of Prussia, PA, USA) was diluted to concentrations of up to 10% (w/v) in 10% sucrose as previously described (Shnitko and Robinson, 2015). Naltrexone (NTX) HCl (Sigma Aldrich) was dissolved in sterile saline. All injections delivered 1 ml/kg volume.

### Operant Acquisition and Maintenance

The experimental timeline is outlined in Table 1. Rats were administered saline or nicotine, with cage-mates assigned the same treatment to minimize differences in behavior within a cage after drug exposure. Starting P28, animals received daily saline or nicotine injections for five consecutive days prior to the first behavior session. Behavioral experiments were performed in 12.0″ × 9.5″ × 8.25″ operant chambers (Med Associates, Inc., St. Albans, VT, USA). Beam break detectors were used to measure a nosepoke response. A cue light was located above the reward delivery well to indicate the availability to retrieve the reward. Animals underwent one session per day for 6–7 days per week. To avoid acute effects of nicotine on operant behavior (Chaudhri et al., 2005), nicotine or saline injections were made 2 h after each training session.

**Table 1:** Experimental timeline.

Age range at start of epoch	Experimental epoch	
P28	Daily nicotine injections for 5 days	
P33	**Operant training**	
	*Sessions 1–3*:	*FR1, 10S (water restricted)*
	*Session 4*:	*FR1, 10S*^a^
	*Sessions 5, 6*:	*FR1, 10S/2.5E*
	*Sessions 7, 8*:	*FR1, 10S/5E*
	*Session 9*:	*FR1, 10S/10E*
	*Sessions 10*:	*FR3, 10S/10E*
	*Sessions 11–21*:	*FR5, 10S/10E*
P57–P61	Naltrexone dose-response sessions	
	Maintenance sessions, FR5, 10S/10E	
P76–P90	Satiety-specific devaluation session	
	Maintenance sessions, FR5, 10S/10E	
P84–P95	Progressive ratio session	
P85–P96	12 extinction sessions	
P97–P108	Reinstatement session	

*^a^4/18 rats did not undergo this session (2 saline and 2 nicotine rats)*.

To encourage acquisition of operant responding for reward, on P34 animals were water restricted the day prior and concurrently with the three initial days of nosepoke training; one nosepoke resulted in concurrent delivery of a cue light and a tone for 3.5 s, 100 μL of reward solution, and a 20 s time out. Nosepoke training consisted of 100 μL of 10% sucrose reward (10S) on an FR1 schedule of reinforcement; each session lasted 2–3 h, immediately followed by home cage access to water for 1 h. After the third session, animals returned to *ad libitum* water access in their home cages for the duration of the study. Next, each phase of training took place during 20–30 min sessions as described in Table 1 (reinforcement schedule-reward): FR1-10S; FR1-10S/2.5E (10% sucrose, 2.5% ethanol, both w/v); FR1-10S/5E; FR1-10S/10E; FR3-10S/10E. The age of first exposure to ethanol (10% sucrose, 2.5% ethanol) was P37–38. Following this schedule, animals were maintained on 30-min sessions of an FR5 schedule of reinforcement with 10S/10E reward. Prior to experimental manipulations below, each animal underwent 11 maintenance sessions on FR5-10S/10E. Note that sweetened alcohol was used to promote rapid acquisition of ethanol seeking during the short period of rodent adolescence (approximately P28–P42).

### Naltrexone

Each animal received 0, 0.3, 1 and 3 mg/kg (SC) NTX 30 min prior to a standard FR5-10S/10E self-administration session. The doses were counterbalanced such that every animal received every dose, but also equal numbers of rats from each group received the same dose order. Animals underwent two maintenance self-administration sessions between NTX sessions to allow wash-out of the prior NTX dose and to re-establish baseline behavior.

### Satiety Specific Devaluation

Next we assessed habitual vs. goal-directed reward seeking with the satiety specific devaluation as previously described (Hay et al., 2013; Shillinglaw et al., 2014), except in the present study we used a between-subjects approach. After the last NTX session, over 3 days leading up to the devaluation session, animal pairs were separated in their home cage by a plastic divider and allowed to drink water from inserted water bottles freely for 60 min prior to standard maintenance sessions; this habituated the rats to the separation and bottle placement. On the test day, animals were separated and allowed to freely drink either 2% maltodextrin solution (control) or 10S/10E solution (devaluation) in a between-subjects design in the 60 min prior to behavioral session. The devaluation test session consisted of a 10-min operant session during which nosepokes were recorded but cue and reward reinforcements were absent.

### Progressive Ratio

Following devaluation, animals underwent 4 days of maintenance self-administration sessions to re-establish baseline behavior. Animals were then assessed for motivation for sweetened ethanol with a progressive ratio schedule of reinforcement as previously described (Walker and Koob, 2007). Animals were placed in the operant conditioning boxes and the number of responses required for reinforcement increased according to the schedule of 1, 1, 2, 2, 3, 3, 4, 4, 5, 5, 7, 7, 9, 9, 11, 11, 13, 13, 15, 15, 18, 18, 21, 21, 24, 24, etc. The session ended after an animal failed to respond for 30 min with a maximum session of 3 h. Nosepokes and “breakpoint” was recorded. The “breakpoint” was the last level of required responding achieved by an animal prior to the end of the session.

### Extinction and Reinstatement

Following the progressive ratio session, with no maintenance sessions between, animals underwent 12 30-min extinction sessions in which they were placed in the operant boxes and nosepokes were recorded but cues and rewards were not delivered. After the last extinction session, animals underwent a 30-min extinction-reinstatement session that began as an extinction session, where cues and reward reinforcements were not delivered. Ten minutes into the session, a single cue delivery consisting of cue light and tone for 3.5 s was delivered. Thereafter, the conditioned (cue) reinforcements were delivered on an FR5 schedule but no reward was delivered, similar to previous studies (Bienkowski et al., 2000; Hay et al., 2013).

### Statistical Analysis

All figures were generated using GraphPad Prism software (GraphPad Software, La Jolla, CA, USA). Statistical analyses were performed using Statistica Software (Statistica, Tulsa, OK, USA). Most experiments were analyzed using two-way ANOVA with session, time bin, or dose as repeated measures and exposure history (saline or nicotine) as the between-subjects factor. Satiety-induced reward devaluation analysis was performed as a two-way ANOVA with no repeated measure. Bonferroni corrected *t*-tests were performed for *post hoc* analyses as appropriate. Progressive ratio analyses were performed as independent sample *t*-tests.

## Results

### Acquisition and Maintenance of Self-Administration of Sweetened Ethanol

We first measured if nicotine exposure beginning during adolescence altered acquisition of operant self-administration of sweetened ethanol. The first four sessions were variable as the rats learned the operant response under water restriction and then were removed from water restriction. Thus, we compared the six sessions in which ethanol was gradually added to the sucrose solution. Nicotine injections 2 h after each behavioral session did not alter acquisition of sweetened ethanol self-administration (Table 2). A two-way repeated measures ANOVA yielded a main effect of session (*F*_(1,5)_ = 7.35, *p* < 0.001), but no significant effect of exposure (*F*_(1,5)_ = 1.37, *p* = 0.26) or session by exposure interaction (*F*_(1,5)_ = 1.56, *p* = 0.18). Collapsed across exposure group, *post hoc* analysis revealed that animals made significantly more nosepokes in the second session on FR1-10S/2.5E than in the first session on that schedule, and that animals made significantly fewer nosepokes in the session on FR1-10S10E than in the previous three sessions (Bonferroni *t*-test, *p* < 0.05).

**Table 2:** Acquisition of operant self-administration, after water restriction.

Session	Saline	Nicotine
FR1, 10S/2.5E	131.9 ± 23.7	82.3 ± 16.7
FR1 10S/2.5E^a^	155.6 ± 14.2	135.3 ± 12.7
FR1, 10S/5E	151.1 ± 18.5	112.8 ± 13.0
FR1, 10S/5E	146.3 ± 20.8	135.4 ± 14.9
FR1, 10S/10E^b^	78.5 ± 11.1	85.2 ± 10.4
FR3, 10S/10E	115.4 ± 21.8	116.2 ± 12.5

*Data shown as mean nosepokes ± SEM. ^a^Collapsed across exposure group, higher than the previous session. ^b^Collapsed across exposure group, lower than the previous three sessions*.

**Figure 1:**
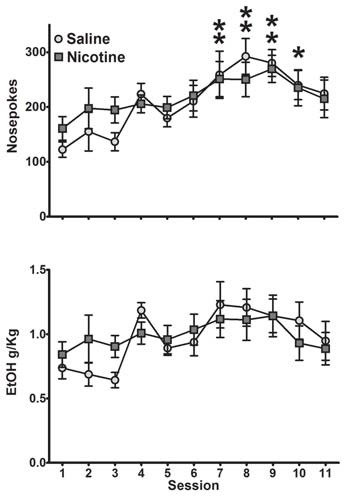
Animals exposed to saline and nicotine self-administered sweetened ethanol similarly. Both nosepokes (Top) and consumption (Bottom) were not significantly different between exposure groups (Bonferroni *post hoc* *t*-test, collapsed across drug exposure groups: *p < 0.05, **p < 0.01 compared to first session; *N* = 8 saline, 10 nicotine).

We next measured whether saline and nicotine groups differed in their maintenance of operant behavior. Saline- and nicotine-exposed animals exhibited similar levels of self-administration as measured by nosepokes and sweetened ethanol consumed (Figure 1). A two-way ANOVA for nosepokes per session revealed a main effect of session (*F*_(10,160)_ = 5.53, *p* < 0.0001), no main effect of exposure (*F*_(1,16)_ = 0.13, *p* = 0.72) and no significant session by exposure interaction (*F*_(10,160)_ = 0.66, *p* = 0.76). Collapsed across exposure group, *post hoc* analysis showed significant increase in nosepokes in sessions 7 through 10 compared to the first session (Bonferroni *t*-test, *p* < 0.05). Similarly, a two-way ANOVA for ethanol consumed per session yielded a significant main effect of session (*F*_(10,160)_ = 3.18, *p* < 0.001), but no main effect of exposure (*F*_(1,16)_ = 0.04, *p* = 0.85), and no significant session-by-exposure interaction (*F*_(10,160)_ = 0.91, *p* = 0.53). Specifically, rats self-administered an average of 0.6–1.2 g/kg ethanol in a session. We also compared body weight during these sessions (data not shown), and detected the expected effect of session (weight gain over days; (*F*_(10,160)_ = 156.3, *p* < 0.001), but no significant effect of exposure (*F*_(1,16)_ = 0.25, *p* = 0.62) or interaction (*F*_(10,160)_ = 0.19, *p* = 0.99). Thus, exposure to nicotine starting in adolescence but administered after the operant sessions had no impact on self-administration of sweetened ethanol.

### Naltrexone Effects on Sweetened Ethanol Seeking

**Figure 2:**
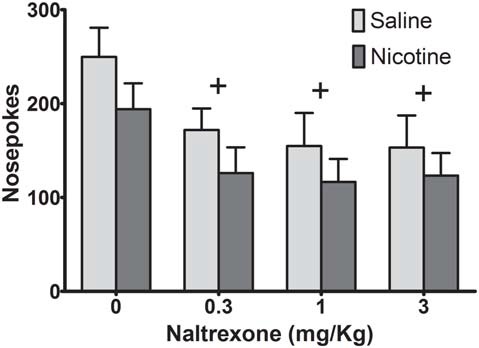
Naltrexone (NTX) reduced operant behavior similarly in saline- and nicotine-exposed rats. While all doses of NTX decreased operant responding, no significant differences in responding were observed between groups (Bonferroni *post hoc* *t*-test, collapsed across drug exposure groups: ^+^*p* < 0.01 compared to vehicle; *N* = 8 saline, 10 nicotine).

To determine if adolescent nicotine exposure affects the ability of NTX to alter ethanol seeking in animals, we administered a dose range of NTX that has been shown to differentially decrease ethanol vs. sucrose self-administration (Czachowski and Delory, 2009; Hay et al., 2013), using a within-subject design (Figure 2). A two-way ANOVA on nosepokes revealed a significant main effect of NTX (*F*_(3,48)_ = 8.03, *p* < 0.001), but no main effect of adolescent drug exposure history (*F*_(1,16)_ = 1.81, *p* = 0.20) and no significant dose-by-exposure interaction (*F*_(3,48)_ = 0.15, *p* = 0.93). *Post hoc* analysis collapsed across drug exposure history indicated significant decreases in sweetened ethanol seeking for all NTX doses compared to control (*p* < 0.01). Therefore, while NTX reduced sweetened ethanol self-administration, exposure to nicotine did not alter NTX’s effects on ethanol seeking.

### Satiety-Specific Reward Devaluation

**Figure 3:**
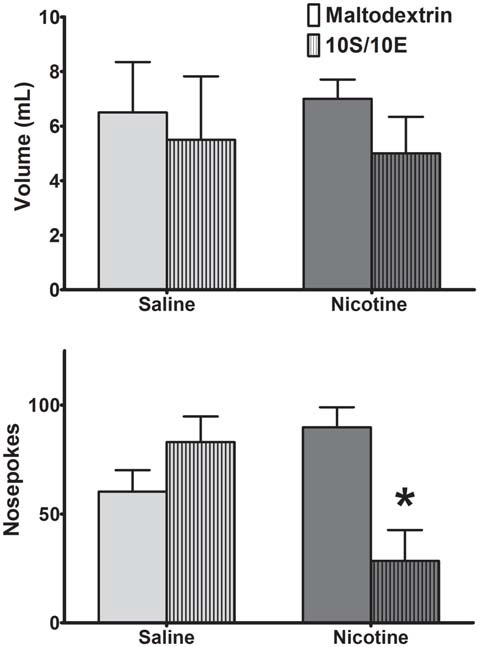
Animals exhibited sustained habitual behavior following reward devaluation except when exposed to nicotine. (Top) Saline and nicotine animals consumed similar volumes of maltodextrin (solid bars) and sweetened ethanol (patterned bars) prior to behavioral measurement. (Bottom) Compared to maltodextrin, a control for consumed volume, pre-exposure to sweetened ethanol altered reward seeking only in nicotine exposed rats (Bonferroni *post hoc* *t*-test: *p < 0.01 compared to maltodextrin access nicotine group; *N* = 4 Maltodextrin-Saline, 4 10S/10E-Saline, 5 Maltodextrin-Nicotine, 5 10S/10E-Nicotine).

Repeated exposure to nicotine can induce habitual nicotine seeking (Clemens et al., 2014). However, it is unknown if this inflexible behavior is generalizable to rewards other than nicotine. Thus, we used satiety-specific reward devaluation to determine if exposure to nicotine also renders an animal more susceptible to habitual behavior. Animals received *ad libitum* access to either 10S/10E or 2% maltodextrin control solution for 60 min prior to a 10 min extinction session (Figure 3). Saline-exposed animals did not alter reward seeking after 10S/10E devaluation compared to rats receiving the 2% maltodextrin control, indicating habitual reward seeking. In contrast, nicotine-exposed rats decreased operant responding following reward devaluation compared to rats under non-devalued conditions. A two-way ANOVA for nosepokes revealed no significant main effect of consumed solution (*F*_(1,14)_ = 2.70, *p* = 0.12), and no significant main effect of drug exposure (*F*_(1,14)_ = 1.14, *p* = 0.30), but a significant solution-by-exposure interaction (*F*_(1,14)_ = 12.83, *p* < 0.01). *Post hoc* analysis resulted in significant decrease in responding after 10S/10E devaluation compared to maltodextrin only in the nicotine-exposed rats (*p* < 0.01). This difference in reward seeking was not due to differences in liquid consumption during the 60-min access period, as there was no significant difference in volume of solution consumed prior to behavioral testing between exposure groups. A two-way ANOVA of liquid consumed prior to behavioral testing revealed no significant main effects of solution (*F*_(1,14)_ = 0.92, *p* = 0.35) and drug exposure (*F*_(1,14)_ < 0.0001, *p* ≈ 1.00), nor a significant solution-by-exposure interaction (*F*_(1,14)_ = 0.10, *p* = 0.75). Though nicotine was expected to induce habitual behavior, nicotine exposed animals were more responsive to reward devaluation, and therefore resistant to habit formation towards sweetened ethanol in this experiment.

### Progressive Ratio

**Figure 4:**
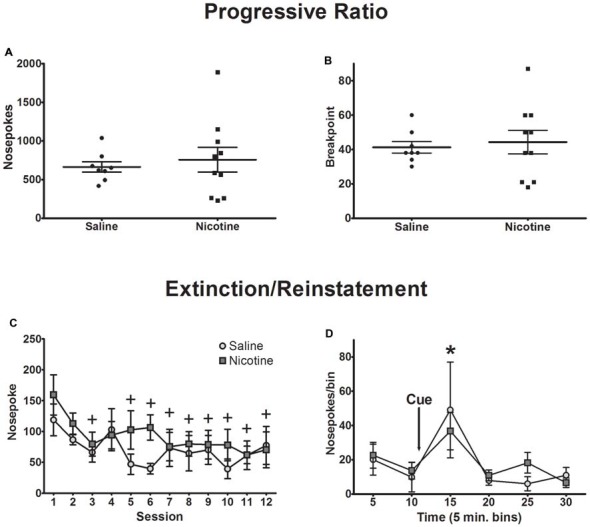
Exposure to nicotine did not affect behavior during progressive ratio, extinction and reinstatement sessions. Motivation towards sweetened ethanol as measured by progressive ratio testing did not differ between exposure groups, indicated by **(A)** total nosepokes and **(B)** behavioral breakpoint. Individual rat data points are presented, with the mean ± SEM overlaid. **(C)** Behavioral responding decreased across 12 extinction sessions similarly between exposure groups. **(D)** Cue-induced reinstatement of operant responding was not different between groups (Bonferroni *post hoc* *t*-test, collapsed across drug exposure groups: ^+^*p* < 0.01 compared to session 1; *p < 0.05 compared to 10, 20, 25 and 30 min bins; *N* = 8 saline, 10 nicotine).

Nicotine can enhance motivation for cue-reward pairing (Chaudhri et al., 2006, 2007). To test whether exposure to nicotine in adolescence alters motivation for sweetened ethanol, we recorded nosepokes and the breakpoint in a progressive ratio session (Figures 4A,B). Independent samples *t*-tests indicated that nicotine had no effect on nosepokes (*t*_(16)_ = 0.49, *p* = 0.63) or breakpoint (*t*_(16)_ = 0.37, *p* = 0.72). Thus, chronic nicotine exposure had no impact on motivation for sweetened ethanol.

### Extinction and Reinstatement of Operant Behavior

Extinction learning was measured over 12 sessions. No significant difference in decreased reward seeking was observed between saline and nicotine exposed animals (Figure 4C). A two-way ANOVA of nosepokes resulted in a main effect of session (*F*_(11,176)_ = 4.33, *p* < 0.0001), but no main effect of drug exposure (*F*_(1,16)_ = 0.59, *p* = 0.45), and no significant session-by-exposure interaction (*F*_(11,176)_ = 1.38, *p* = 0.18). Nosepokes decreased significantly in the last eight sessions of extinction as indicated by *post hoc* analysis collapsed across drug exposure groups (*p* < 0.01).

As exposure to nicotine during adolescence has been shown to increase responding for conditioned reinforcers when compared to saline-exposed animals (Quick et al., 2014), we measured reinstatement of reward seeking after exposure to reward-associated cues. Reinstatement of operant responding for the cues previously associated with sweetened ethanol was measured in a single extinction-reinstatement session. Presentation of the cue (light and tone) 10 min into the session resulted in a significant increase in responding, but no significant difference was found between saline- and nicotine-exposed animals (Figure 4D). A two-way ANOVA of nosepokes across 5-min bins revealed a significant main effect of time bins (*F*_(5,80)_ = 4.64, *p* < 0.001), but no significant main effect of drug exposure (*F*_(1,16)_ = 0.01, *p* = 0.92) or significant time-by-exposure interaction (*F*_(5,80)_ = 0.46, *p* = 0.80). *Post hoc* analysis collapsed across drug exposure groups showed that cue presentation was sufficient to induce reinstatement of reward seeking, as nosepokes in the 15-min, post-cue time bin was significantly higher than the 10-min, pre-cue time bin (*p* < 0.05). Collectively, these data show that nicotine exposure history had no impact on extinction of reward seeking or reinstatement of responding after re-exposure to reward-associated cues.

## Discussion

Epidemiological studies find that initiating nicotine consumption in adolescence correlates with progression to escalating use as well as to the propensity to pursue more illicit substances of abuse (Kandel and Faust, 1975; Kandel et al., 1992). The Gateway Hypothesis of addiction suggests that neurophysiological changes due to nicotine exposure would eventually lead to escalation of drug use due to increased reward valence to substances of abuse (Kandel and Kandel, 2014). Previous findings suggest exposure to nicotine only in adolescence is insufficient to alter adult cocaine self-administration (Pomfrey et al., 2015). Indeed, the escalation of use and progression to stronger drugs may rely on continuous drug exposure throughout adolescence as opposed to priming only (Kandel and Kandel, 2015). Given the propensity of nicotine and ethanol use in adolescence, it is also important to determine if nicotine can be a gateway drug to alcohol use. We hypothesized that animals exposed to daily injections of nicotine beginning in adolescence would be more sensitive to the rewarding and reinforcing properties of sweetened ethanol, and therefore would increase seeking and motivation for ethanol in a self-administration paradigm. However, as discussed below, our findings did not support this hypothesis.

Studies show that co-administration of nicotine increases alcohol seeking (Smith et al., 1999; Lê et al., 2014; Lárraga et al., 2017) and VTA dopamine neuron activity in rodents (Tolu et al., 2017). However, this may be due to nicotine’s well-known ability to enhance the reinforcing properties of conditioned cues (Donny et al., 2003; Olausson et al., 2004a,b; Chaudhri et al., 2006; Palmatier et al., 2007, 2013; Caggiula et al., 2009; Guy and Fletcher, 2014a,b; Yager and Robinson, 2015). Indeed, several studies reported that nicotine administered prior to each behavioral session elevated conditioned responding to cues predicting either sucrose (Palmatier et al., 2013; Stringfield et al., 2017) or ethanol (Maddux and Chaudhri, 2017), supporting the contention that nicotine enhances approach behavior. Moreover, presentation of a nicotine-associated context, and not necessarily in the presence of nicotine, can increase ethanol self-administration (Zipori et al., 2017), suggesting the possibility that nicotine-enhancement of contextual cues is what actually drives enhanced ethanol seeking. Therefore, nicotine administered outside of the behavioral context may not alter self-administration, which is somewhat inconsistent with a molecular basis of the Gateway Hypothesis of nicotine (Kandel and Kandel, 2014). To test this, we gave adolescent rats daily injections of nicotine for 5 days prior to the start of behavioral training, and then, every day of behavioral training, animals received nicotine 2 h after the behavioral session. In short, rats received nicotine such that there was no association with the behavioral sessions and minimal pharmacological interaction with the ethanol consumed. We found that this nicotine regimen resulted in no significant impact on self-administration of sweetened ethanol; therefore, these results do not support a strictly “exposure” interpretation of the Gateway Hypothesis of nicotine. However, it should be noted that preclinical studies supporting the Gateway Hypothesis used a continuous exposure model of nicotine in drinking water (Levine et al., 2011), whereas we administered a single bolus of nicotine per day.

NTX is an opioid receptor antagonist that reduces ethanol seeking and the rewarding properties of ethanol (e.g., Kreek et al., 2002; Ripley and Stephens, 2011). We chose a dose range of NTX that has been shown to decrease ethanol seeking with nominal effect on sucrose seeking (Czachowski and Delory, 2009; Hay et al., 2013). NTX is more effective in reducing ethanol use in cigarette smokers compared to nonsmokers (Fucito et al., 2012) and in animals co-administering nicotine and ethanol during self-administration compared to ethanol alone (Lê et al., 2014). However, we found that NTX decreased sweetened ethanol seeking similarly in animals exposed to nicotine and saline. Our study was performed in a peri-adolescent period of development (~P60), whereas most studies showing nicotine promotion of ethanol drinking have been done in adults. However, there is no evidence suggesting that NTX lacks efficacy in curbing ethanol seeking during adolescence. Indeed, NTX has been shown to decrease ethanol seeking in adolescent humans (Deas et al., 2005) and rodents (Sable et al., 2006). Thus, while previous findings show that NTX is a viable tool to decrease ethanol seeking in patients with a history of smoking, our results suggest this is likely due to nicotine-enhanced seeking behavior as opposed to nicotine exposure *per se*. As discussed above, our nicotine administration paradigm was such that animals were not exposed to nicotine in the context of the behavioral paradigm or in tandem with ethanol. As such, the cue reinforcing effects of nicotine (e.g., Caggiula et al., 2009) were likely not contributing factors to the results observed here.

Habit-directed behavior is a form of behavioral inflexibility, in that an animal is less likely to alter habitual behavior following a change in reward value. Ethanol consumption can shift operant behavior from goal- to habit-directed (Dickinson et al., 2002; Corbit et al., 2012; Mangieri et al., 2012). In turn, adolescent ethanol exposure can also lead to reduced behavioral flexibility in adulthood (Coleman et al., 2014; Gass et al., 2014; Madayag et al., 2017). Therefore, it is not surprising that the control animals in our study exhibited heightened habit-directed alcohol seeking in a reward devaluation paradigm, although extended operant training can also be sufficient to induce habit formation (e.g., Ostlund and Balleine, 2008). Repeated use of nicotine leads to habitual nicotine-seeking behavior (Clemens et al., 2014; Loughlin et al., 2017), though this can depend on the number of prior self-administration sessions, as 47 sessions, but not 10 sessions, produced insensitivity to nicotine devaluation (Clemens et al., 2014). Thus, one may expect that rats exposed to nicotine over 40 days would be predisposed to habitual reward seeking, but that is not what we observed in the present study. In fact, animals exposed to nicotine exhibited sensitivity to reward devaluation compared to animals that received saline control. This is not likely due to acute effects of nicotine as the latest injection of nicotine was approximately 22 h prior to the behavioral session. Alternatively, it is possible that the ability of nicotine to maintain goal-directed behavior towards sweetened ethanol seeking was due to long-term exposure, as chronic exposure to nicotine can result in different nicotinic receptor subunit expression. For example, when administered in adolescence, repeated nicotine increases β2 subunit-containing nicotinic receptors (Counotte et al., 2012a). In turn, compounds that selectively target β2 subunit-containing receptors can enhance behavioral flexibility in humans and primates withdrawn from drugs of abuse (Gould et al., 2013; Terry et al., 2016; Lesage et al., 2017). Therefore, the administration of nicotine outside of the behavioral context may have enhanced behavioral flexibility and, therefore, rendered animals in our study resistant to habit-directed behavior.

Contingent nicotine co-administered with ethanol and non-contingent nicotine administered prior to ethanol self-administration sessions increase the motivation for ethanol seeking both in rodents (Bespalov et al., 1999) and humans (Barrett et al., 2006). When administered in conjunction with self-administered ethanol, nicotine enhances the motivation for ethanol in dependent rats (Leão et al., 2015). Consistent with this, administration of nicotine vs. non-nicotine cigarettes in human smokers increased the breakpoint for alcoholic beverages (Barrett et al., 2006). However, we observed no effect on motivation (nosepokes, breakpoint) for sweetened ethanol in animals administered nicotine when the nicotine was given outside of the behavioral context. This indicates that the motivationally enhancing effects of nicotine on ethanol seeking is likely to be highly dependent on the temporal aspect of administration.

Nicotinic receptor agonists including nicotine can be used to enhance cognition and behavioral flexibility (for review see Counotte et al., 2012b). For example, when given after induction of fear conditioning, nicotine has been shown to enhance extinction learning (Elias et al., 2010). Therefore, we expected animals exposed to nicotine to exhibit faster extinction from sweetened ethanol seeking compared to animals exposed to saline. Contrary to this prediction, we observed no significant difference in extinction behavior between the two groups, suggesting that the cognitive-enhancing effects of nicotine did not extend to extinction learning in the present study. Furthermore, when administered during ethanol withdrawal, nicotine can increase reinstatement of ethanol self-administration (López-Moreno et al., 2004). However, we observed no differences in reinstatement behavior after re-exposure to the associated cue. This likely occurred because we avoided acute nicotine effects on the reinstatement session, consistent with previous reports in which a temporally distant exposure had no effect on reinstatement behavior (Hauser et al., 2012).

Synergistic or additive effects on reinforcement circuitry due to co-administration or co-consumption of nicotine and ethanol (Leão et al., 2015; Tolu et al., 2017) are unlikely to contribute to the present results. The half-life of available nicotine in the brain is approximately 52 min (Ghosheh et al., 1999). Therefore, sufficient pharmacologically active nicotine should be available during the behavior session when administered up to 3–4 h prior (Hauser et al., 2012) to render behavioral effects from an acute injection, but not likely when administered approximately 22 h prior to the behavior session as in the present study. On the other hand, Doyon et al. (2013) reported long-term effects of nicotine on ethanol-evoked dopamine levels, lasting up to 40 h. Thus, the lack of effect of nicotine on behavior in the present study is consistent with expected pharmacokinetics, and while it is possible that this nicotine regimen produced persistent effects on the dopamine system, they were evidently insufficient to alter sweetened ethanol self-administration in the present study.

One caveat to the present study is that it used only female rats, as ethanol self-administration extended from mid-adolescence into adulthood, and female rodents are well-known to drink more ethanol than males in adulthood (Becker and Koob, 2016). However, in adolescence the sex difference is less clear, as some studies found that males drank more ethanol than females (e.g., Vetter-O’Hagen et al., 2009) and others found more drinking in females (e.g., Varlinskaya et al., 2015). When administered only during adolescence, nicotine enhanced conditioned reinforcement in both male and female adults, but increased Pavlovian conditioned approach only in adult males (Quick et al., 2014). Thus, it is possible that nicotine would have different effects in males under the current study design, which may be addressed by future studies.

A related issue is that behavioral neuroscience has historically largely used male rodents based on the assumption that females would introduce greater day-to-day variability due to hormonal effects across their estrous cycle (McCarthy, 2015; Guizzetti et al., 2016). Thus, it is possible that the present study “missed” effects of nicotine due to hormone-related variability in female self-administration. However, recent studies analyzing published data found this assumption to be erroneous: in general, female rodents exhibit no different variance in most metrics (physiological, behavioral, histological, etc.) compared to males (Prendergast et al., 2014; Becker et al., 2016). Even for those traits that showed sex differences, female data were not inherently more variable than male data (Becker et al., 2016). In humans, the primary discrepancy between sexes is that women metabolize nicotine faster than men (Benowitz et al., 2006), although menstrual cycle has no impact on the rate of metabolism in human subjects (Hukkanen et al., 2005). Collectively, it appears unlikely that using only females inserted more variability in behavior metrics. However, future studies that directly compare the effects of nicotine on ethanol self-administration in males and females are needed to determine sex differences in day-to-day variability of intake and the potential effects of nicotine.

According to the Gateway Hypothesis of addiction, prior exposure to nicotine leads to an increase in sensitivity to the rewarding properties of other substances of abuse (Kandel and Kandel, 2014). We observed that initiating nicotine administration during adolescence and continuing into adulthood was insufficient to increase ethanol seeking. Therefore, it is likely that nicotine must be self-administered or associative cues must be present while experimenter-administered nicotine is physiologically active for it to produce its reward-amplifying properties. The Gateway Hypothesis should not be discounted, but further investigation should aim to determine in what capacity “gateway drugs” contribute to progression to and escalation of consumption of substances of abuse.

## Author Contributions

ACM designed the experimental methods and was the primary contributor to authoring the manuscript. KSC performed the experiments and was a secondary contributor to authoring the manuscript. LMW performed the experiments and reviewed the manuscript. DLR helped design the experimental method, reviewed the manuscript and is the principal investigator.

## Conflict of Interest Statement

The authors declare that the research was conducted in the absence of any commercial or financial relationships that could be construed as a potential conflict of interest.
